# Aligned chitosan nanofiber hydrogel grafted with peptides mimicking bioactive brain-derived neurotrophic factor and vascular endothelial growth factor repair long-distance sciatic nerve defects in rats

**DOI:** 10.7150/thno.36272

**Published:** 2020-01-01

**Authors:** Feng Rao, Yanhua Wang, Dianying Zhang, Changfeng Lu, Zheng Cao, Jiajie Sui, Mengjiao Wu, Yawen Zhang, Wei Pi, Bo Wang, Yuhui Kou, Xiumei Wang, Peixun Zhang, Baoguo Jiang

**Affiliations:** 1 Department of Orthopedics and Trauma, Peking University People's Hospital, Beijing100044, China; 2 State Key Laboratory of New Ceramics and Fine Processing, School of Materials Science and Engineering, Tsinghua University, Beijing 100084, China; 3 Key laboratory of Carcinogenesis and Translational Research (Ministry of Education/Beijing), Laboratory of Molecular Oncology, Peking University Cancer Hospital & Institute, Beijing 100142, China; 4 Department of Orthopedics, Peking University Third Hospital, Beijing100191, China.

**Keywords:** Peripheral nerve injury, electrospinning, mechanical stretching, chitosan nanofibers, peptides.

## Abstract

Autologous nerve transplantation, which is the gold standard for clinical treatment of peripheral nerve injury, still has many limitations. In this study, aligned chitosan fiber hydrogel (ACG) grafted with a bioactive peptide mixture consisting of RGI (Ac-RGIDKRHWNSQGG) and KLT (Ac-KLTWQELYQLKYKGIGG), designated as ACG-RGI/KLT, was used as nerve conduit filler to repair sciatic nerve defects in rats.

**Methods**: Chitosan nanofiber hydrogel was prepared by a combination of electrospinning and mechanical stretching methods, and was then grafted with RGI and KLT, which are peptides mimicking brain-derived neurotrophic factor (BDNF) and vascular endothelial growth factor (VEGF), respectively. The physicochemical properties of ACG-RGI/KLT were fully characterized. *In vitro*, the distribution, proliferation, and secretory activity of Schwann cells were analyzed. Next, the *in vivo* repair potential for 15-mm rat sciatic nerve defects was examined. The recovery of regenerated nerve, muscle, and motor function was evaluated by neuromuscular histology, electrophysiology, and catwalk gait analysis.

**Results**: We first constructed directionally aligned chitosan nanofiber hydrogel grafted with RGI/KLT peptide mixture (ACG-RGI/KLT). ACG-RGI/KLT oriented the Schwann cells, and promoted the proliferation and secretion of neurotrophic factors by Schwann cells. At an early injury stage, ACG-RGI/KLT not only enhanced nerve regeneration, but also promoted vascular penetration. At 12 weeks, ACG-RGI/KLT facilitated nerve regeneration and functional recovery in rats.

**Conclusions**: Aligned chitosan nanofiber hydrogel grafted with RGI/KLT peptide provides an effective means of repairing sciatic nerve defects and shows great potential for clinical application.

## Introduction

The increasing frequency of traffic accidents due to the rapid development of modern traffic systems, as well as major public health incidents such as earthquakes and explosions, have led to an increase in the number of patients with peripheral nerve injury 1-5. At present, autologous nerve grafting is still the gold standard for treatment of peripheral nerve defects 6. However, autologous nerve grafting has many limitations, such as insufficient graft sources and painful neuroma. Therefore, artificial nerve grafts are considered as a promising alternative to autologous nerve grafting 6-8.

Tissue-engineered nerve grafts could provide a suitable microenvironment for nerve regeneration 7, 9. Many nerve grafts have been developed in the form of nerve guidance conduits (NGCs) 10-13. The material sources of NGCs mainly consist of natural macromolecules, such as chitosan, collagen, and silk fibroin, as well as synthetic polymer materials such as polylactic acid, polylactic-co-glycolic acid (PLGA), and polycaprolactone (PCL) 6, 14. However, hollow NGCs do not fulfill the requirements for nerve regeneration. To maximize nerve regeneration, many improvements have been made based on hollow conduits, such as modifying the inner wall of the conduit 15-18, constructing nerve conduits with multiple channels 7, 19-21 and adding fillers to the conduit. Among these, fillers have been studied in the greatest detail; they include porous hydrogels 22-24, sponge-like porous scaffolds 25, and aligned fibrin hydrogels 26. These fillers mainly simulate the extracellular matrix (ECM) to provide a suitable microenvironment for nerve regeneration. Among the nerve conduit fillers, fibers with an aligned structure can best simulate the anatomical structure and directional arrangement of nerve fibers. Hydrogel, a type of three-dimensional network polymer with a water content of 80%, has good wettability and biocompatibility. However, many natural hydrogel matrices are limited by their intrinsically chaotic structure.

Electrospinning is a classical, controllable nanofiber production technology that can simulate the microstructure of the ECM and has become a common technique for preparing tissue engineering scaffolds, including skin dressing 27, 28, cartilage and bone tissue engineering 29, 30, tendon tissue engineering 31, 32, and nerve tissue engineering 25, 33. However, given the many natural materials available, it is important to select a biocompatible and biodegradable material.

Chitosan is widely used in tissue engineering due to its excellent properties, including antibacterial activity, biocompatibility, non-toxicity, and biodegradability 34-39. Early introduction of exogenous growth factors has been shown to contribute to the repair of injuries, and the addition of appropriate factors to nerve conduits can support the repair of long-distance nerve defects. However, the instability and short half-life of growth factors limit their application in nerve tissue engineering. Therefore, it is necessary to explore effective means of adding growth factors to tissue-engineered nerve grafts. Short peptide fragments with cell-specific functions can mimic the functions of growth factors and significantly improve the biological function and specificity of materials. Previous studies showed that the bioactive RGI peptide (Ac-RGIDKRHWNSQGG), derived from brain-derived neurotrophic factor (BDNF), plays important roles in motor neuron outgrowth 40. Angiogenesis also plays an important role in nerve regeneration. Vascular endothelial growth factor (VEGF) is one of the most important factors in the process of angiogenesis. The peptide KLT (Ac-KLTWQELYQLKYKGIGG) simulates the functional segment of VEGF and acts as an analog. KLT is the only synthetic peptide that can activate the VEGF receptor 41. Therefore, the combination of KLT and RGI may promote microcirculation reconstruction and recovery of motor function.

In this study, aligned chitosan fiber hydrogel (ACG) scaffolds were fabricated by liquid electrospinning and mechanical stretching. Then, biologically active RGI/KLT peptide was grafted onto the chitosan fibers (designated as ACG-RGI/KLT) to build a composite nerve scaffold to repair 15-mm sciatic nerve defects in rats.

## Methods

### Preparation of NGC containing ACG-RGI/KLT

**Figure 1:**
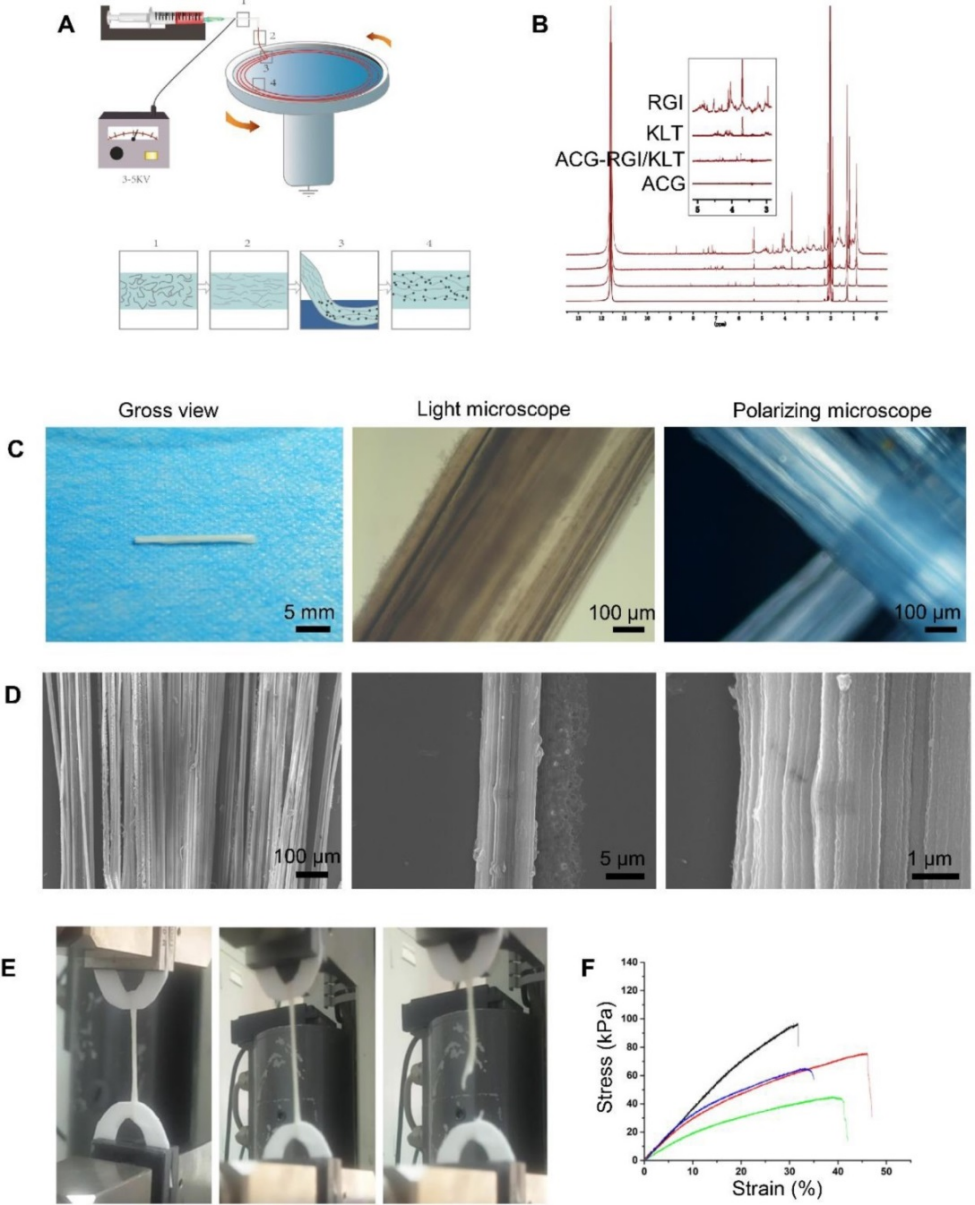
Preparation and characteristics of aligned chitosan fiber hydrogel. (A) Illustration of the electrospinning and mechanical stretching setup. A voltage of 3-5 kV was applied between the spinning solution and collector. Chitosan polymer chains became increasingly aligned as the chitosan hydrogel was extruded from the needle (Box 1) and stretched during entry (Box 2) into a rotating bath. As the chitosan hydrogel entered the rotating bath (Box 3) containing sodium tripolyphosphate (STPP; black dots) under mechanical stretching, chitosan chains were further aligned and crosslinked by STPP in the bath (Box 4), thus forming a stable chitosan nanofiber hydrogel. (B) Proton nuclear magnetic resonance (NMR) spectroscopy of RGI and KLT. (C) Gross view and light microscopic images of an aligned chitosan hydrogel bundle. Polarized light microscopic images showing optical extinction in the crossover region of two chitosan fiber bundles. (D) Scanning electron microscopy (SEM) images of aligned chitosan fiber hydrogel (ACG) showing hierarchically aligned structures under different magnifications. (E) Test of the mechanical properties of aligned chitosan hydrogels. (F) Elastic modulus of aligned chitosan hydrogels.

Chitosan, poly (ethylene glycol) (average MW ca. 4,000 kDa) and sodium tripolyphosphate (STPP) were purchased from Sigma-Aldrich. The aligned chitosan nanofiber hydrogel (ACG) was fabricated by electrospinning as reported previously 26, 42, 43. Briefly, 2% electrospinning chitosan solution was prepared by dissolving 0.2 g of chitosan powder with 10 mL of 2% acetic acid. Then, 0.2% polyethylene oxide (PEO) was added to the electrospinning solution to improve its viscosity. The polymer solution was mixed for 30 min on a rotary mixer until the liquid was evenly mixed. During the electrospinning process, polymer solution was ejected through a 2.5 mL syringe needle at a rate of 3 mL/h, and charged with 4 kV positive potential to form a solution jet. A circular collector was used to collect the aligned chitosan nanofibers at a speed of 60 rpm with 1% STPP aqueous solution (Figure 1A). The chitosan nanofibers were collected and washed in sterile phosphate-buffered saline (PBS) for 6 h to remove the PEO and STPP, followed by immersion in 75% ethanol for 24 h.

The functional polypeptides RGI and KLT were synthesized by solid-phase technology by Shanghai Qiangyao Biotechnology Co., Ltd. High-performance liquid chromatography (HPLC) was performed to verify that the polypeptides had > 95% purity. Samples of 2.5 mg RGI and 2.5 mg KLT polypeptide powders were dissolved in 25 mL of ultrapure water. 1-Ethyl-3-(3-dimethylaminopropyl)carbodiimide (EDC) and *N*-hydroxysuccinimide (NHS) were added to COOH:EDC:NHS in a molar ratio of 10:10:1. The chitosan nanofibers hydrogel grafted RGI and KLT (ACG-RGI/KLT) was constructed by immerse chitosan nanofibers hydrogel into the above solution containing RGI and KLT on shaking bed for 24 h.

The chitin conduits, patented by Peking University People's Hospital and Textile Science Institute of China (Patent No. 01136314), were prepared as described previously 44, 45. NGCs containing ACG or ACG-KLT/RGI were fabricated by inserting ACG or ACG-KLT/RGI into the lumen of the NGCs.

### Polarized light microscopic observation

The chitosan hydrogel obtained by electrospinning was cut into small segments (~5 cm in length) for observation under a polarizing microscope (UPT203i; Leica). First, the polarizer was adjusted in the orthogonal state to achieve complete extinction of the dark field of vision. Then, bunches of chitosan hydrogels were placed in the center of the object stage for imaging. Two bunches of chitosan hydrogels were superimposed at 90° and placed in the center of the object stage for imaging.

### Scanning electron microscopy (SEM) analysis

Chitosan hydrogel was fixed with glutaraldehyde for 30 min and then dehydrated through a graded acetone series (30%, 50%, 70%, 90%, and 100%); this was followed by critical point drying. Samples were imaged by SEM (6700F; JEOL).

### Examination of mechanical properties

The mechanical properties of chitosan hydrogel were measured using a material testing machine (ZwickRoell). Briefly, the two ends of the chitosan hydrogel were fixed on the clamp, and then stretched at a rate of 1 mm/min. The Young's elastic modulus of the chitosan hydrogel was determined from the linear slope of the strain as < 5%, and the stress at gel fracture was taken as the tensile strength. The corresponding strain was defined as the fracture strain.

### Isolation and culture of Schwann cells

Five Sprague-Dawley rats were used to obtain cultures of primary Schwann cells within 3 days after birth, as reported previously 46. Briefly, the bilateral sciatic nerves of the rats were collected after sterilization. The sciatic nerve epicardial membrane was then peeled off using microforceps, and the sciatic nerve was cut into 1-mm segments, enzymatically dissociated in 0.2% collagenase NB4 (17454; SERVA) for 20 min at 37 °C, centrifuged, and resuspended in Dulbecco's modified Eagle's medium (DMEM)/F-12 (Gibco) containing 10% fetal bovine serum (FBS) (10099141; Gibco). The Schwann cells were then seeded in culture dishes and cultured with DMEM/F-12 containing 10% FBS, 2 mM forskolin, and 2 ng/mL heregulin.

### RT-PCR and quantitative PCR

RNA was extracted from Schwann cells in all groups using TRIzol reagent (Invitrogen). cDNA was synthesized using a Synthesis Kit (Roche). Real-time PCR was performed using SYBR Green PCR master mix (Roche) with the forward and reverse primers listed in Table 1. To analyze changes in gene expression, the results of three replicates in three independent experiments were averaged. Levels of gene expression were normalized relative to glyceraldehyde 3-phosphate dehydrogenase (GAPDH).

### Western blotting (WB)

WB was performed using antibodies to VEGF (ab32152), nerve growth factor (NGF; ab6199), BDNF (ab108319), glial cell-derived neurotrophic factor (GDNF; ab18956), neural cell adhesion molecule 1 (NCAM1; ab9018), and growth associated protein 43 (GAP43; ab12274) purchased from Abcam, and to proliferating cell nuclear antigen (PCNA; #13110), protein kinase B (AKT; #4691), phosphorylated protein kinase B (p-AKT; #4060), CD31 (#77699), and GAPDH (#51332) purchased from Cell Signaling Technology.

Schwann cells and sciatic nerves were harvested from the experimental and control groups for WB. Briefly, Schwann cells were lysed in radioimmunoprecipitation assay (RIPA) buffer containing protease inhibitors (Applygen) for protein extraction. Then, aliquots of 20 μg of protein were separated by sodium dodecyl sulfate-polyacrylamide gel electrophoresis (SDS-PAGE) and transferred onto polyvinylidene difluoride (PVDF) membranes (Millipore), which were then incubated with antibodies as described above. Western blots were imaged using an Amersham Imager 600.

### Surgical process

**Figure 2:**
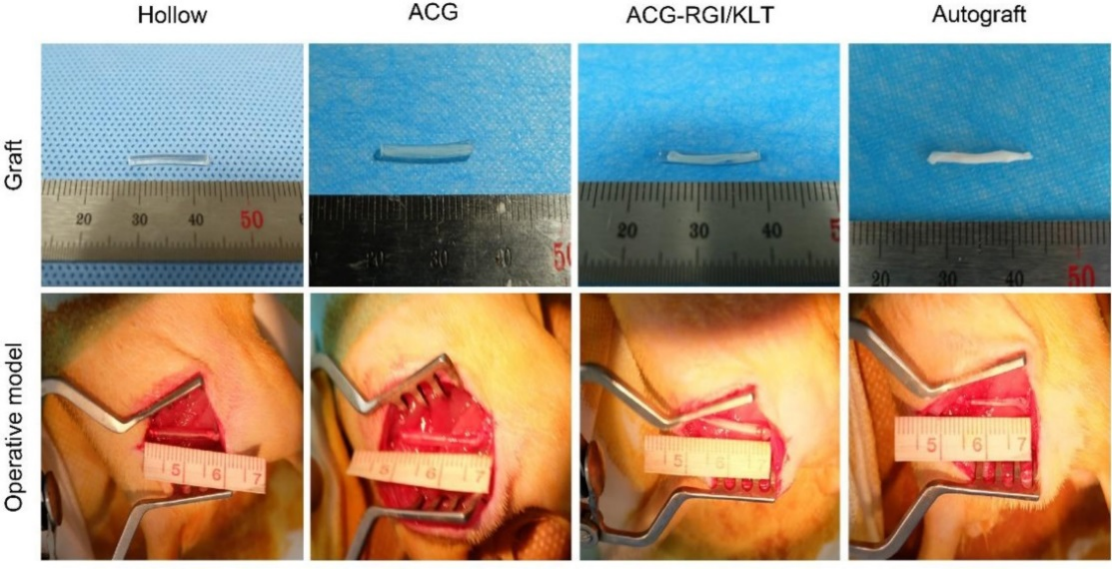
Gross view of nerve grafts and operative model of all groups. The chitin conduits were light yellow in color and transparent. ACG and ACG-RGI/KLT were white, similar to sciatic nerves. All groups received the 15-mm sciatic nerve defect.

Sprague-Dawley rats weighing 200-220 g were purchased from Beijing Vital River Laboratory Animal Technology Co., Ltd. Animals were treated in accordance with the Laboratory Animal Guideline for Ethical Review of Animal Welfare of China (GB/T 35892-2018). All experiments were performed in compliance with the relevant regulations laid out by the Medical Ethics Committee of Peking University People's Hospital. Rats were anesthetized by intraperitoneal injection of 1% sodium pentobarbital solution (30 mg/kg body weight) and randomly divided into four groups: hollow NGC group, NGC containing ACG group, NGC containing ACG-KLT/NGI group, and autograft group. The sciatic nerves of the right hind limbs of rats were cut to leave a 15 mm gap defect. The nerve stumps were bridged with nerve grafts as described above, under a microscope using 10-0 sutures (Figure 2).

### Immunofluorescence

After culture for 5 days, Schwann cells were fixed with paraformaldehyde, washed with PBS, and incubated for 2 h at 37°C with rabbit anti-S100 antibody (S2644; Sigma-Aldrich). After rinsing with PBS, samples were incubated with goat anti-rabbit IgG H+L (Alexa Fluor 594) (ab150084; Abcam) in the dark for 1 h at room temperature. The samples were then rinsed with PBS and counterstained with 4′,6-diamidino-2-phenylindole (DAPI; Solarbio) for 10 min. Regenerated sciatic nerves were cut after 4 weeks, fixed with paraformaldehyde, washed with PBS, incubated for 2 h at 37°C with mouse anti-NF200 antibody (N0142; Sigma-Aldrich) and rabbit anti-CD31 antibody (ab182981; Abcam), and incubated with goat anti-rabbit IgG H+L (Alexa Fluor 594) (ab150084; Abcam) and goat anti-mouse IgG H+L (Alexa Fluor 488) (ab150117; Abcam) in the dark for 1 h at room temperature. The samples were then rinsed with PBS and counterstained with DAPI (Solarbio) for 10 min.

### Cutting of ultrathin sections and transmission electron microscopy (TEM)

Regenerative nerves in all groups were removed after 12 weeks, fixed in 2.5% glutaraldehyde overnight, post-fixed by immersion in 1% osmium tetroxide solution for 3 h, and dehydrated through a graded acetone series (30%, 50%, 70%, 90%, and 100%). The samples were then embedded in Epon 812 epoxy resin and cut into 800 nm semithin sections and 80 nm ultrathin sections with an ultramicrotome. The 800 nm semithin sections were stained with hematoxylin and eosin (HE) and toluidine blue. The 80 nm ultrathin sections were stained with 3% uranyl acetate-lead citrate and examined by TEM. Remyelinated nerve fiber diameter and myelin sheath thickness were determined using Image Pro Plus 6.0 software.

### Muscle evaluation

Gastrocnemius muscles were removed and their wet weight ratios were measured, followed by fixation in 4% paraformaldehyde overnight. Muscle samples were embedded in paraffin, cut into sections 7 μm thick, and subjected to Masson trichrome staining. Photographs were analyzed quantitatively using Image Pro Plus 6.0 (Media Cybernetics).

### Fluoro-gold (FG) retrograde tracing

The regenerative sciatic nerves of rats were re-exposed and cut as reported previously 47. Briefly, the distal end of the regenerated nerve was immersed in 4% FG (Thermo Fisher) for 2 h. The rats were then returned to their cages for 7 days to allow the retrograde tracers to migrate to motor neurons in the anterior horn of the spinal cord and dorsal root ganglion (DRG). After perfusion of the rats with 4% paraformaldehyde, the lumbar spinal cord (L4-6) including all of the sciatic motor neurons and DRG was removed and post-fixed for several hours in 4% paraformaldehyde, followed by cryoprotection in 20% sucrose overnight, and then cut into frozen sections.

### Sciatic nerve index analysis

A CatWalk XT gait analysis system (Noldus), which automatically records movement, was used to analyze the motor function of the rats. First, the camera was adjusted to the appropriate position. Before recording, rats were familiarized with the test environment. The sciatic function index (SFI) was measured by the Brain formula: SFI = 109.5 (ETS - NTS)/NTS - 38.3(EPL - NPL)/NPL + 13.3(EIT - NIT)/NIT - 8.8.

### Evaluation of the electrical conduction of regenerative nerves

The gastrocnemius muscle and sciatic nerve from the operated side of the rats were subjected to electrophysiological evaluation. A Medelec Synergy electrophysiological system (Oxford Instrument Inc.) was used to measure the electrical activity. The stimulating electrodes were placed at the proximal and distal ends of the nerve, while the recording electrodes were placed on the gastrocnemius muscle. The stimulation voltage was adjusted to 20 mV, and the to 1 Hz.

**Statistical analysis.** The data were analyzed using SPSS software (ver. 17.0; SPSS Inc.) with one-way ANOVA followed by Tukey's post hoc multiple comparison test. The results were shown with mean ±SEM. In all analyses, *p*<0.05 was taken to indicate statistical significance.

## Results

### Characterization of ACG-RGI/KLT

In the electrospinning process, the chitosan/PEO fibers were dropped into a rotating collector containing STPP aqueous solution, and a series of changes occurred in the arrangement of the molecules in the electrospinning liquid (Figure 1A). The chitosan molecules were randomly dispersed in the polymer solution. Under the action of the electric field, the molecules adopted a particular directional arrangement. After coming into contact with the receiving liquid in the mechanical rotating disc, the molecules underwent crosslinking reaction and were fixed by the pull of the mechanical rotating disc. The PEO and STPP aqueous solution could be removed easily by dissolving the chitosan/PEO fibers in water.

Proton nuclear magnetic resonance (NMR) spectroscopy (Figure 1B) showed two strong peaks at 2.04 and 11.65 ppm, corresponding to deuterated acetic acid. The pure chitosan hydrogel had relatively strong peaks at 2.04 and 11.65 ppm, with almost no additional peaks. ACG-RGI/KLT showed obvious peaks between 3 and 8 ppm, which were consistent with the peaks of RGI and KLT, respectively.

As shown in Figure 1C, the as-prepared chitosan hydrogel was white, and tightly packed in bundles. Under optical microscopy, the chitosan hydrogel bundles were composed of a large number of micron-scale hydrogel fibers, which also had good orientation. Polarized light microscopy showed obvious optical extinction at the intersection of two vertical chitosan hydrogel fibers. SEM further illustrated the orientation of chitosan hydrogel fibers; low-power images revealed a uniform size of the fibers. High-power images showed the surface details and nanoscale topology of single chitosan fibers (Figure 1D).

The elastic modulus of chitosan hydrogel was examined using a material testing machine (Figure 1E). According to the force-displacement curve, the elastic modulus of the sample was calculated via fitting to the Hertz model. The results showed that chitosan hydrogel had an elastic modulus of 3.10 ± 0.81 kPa, tensile strength of 70.66 ± 22.05 kPa, and strain of 39.46 ± 7.20% (Figure 1F). The elastic modulus of chitosan hydrogel was close to that of nerve tissue, making it suitable for nerve tissue repair.

### ACG-RGI/KLT regulates Schwann cells *in vitro*

**Figure 3:**
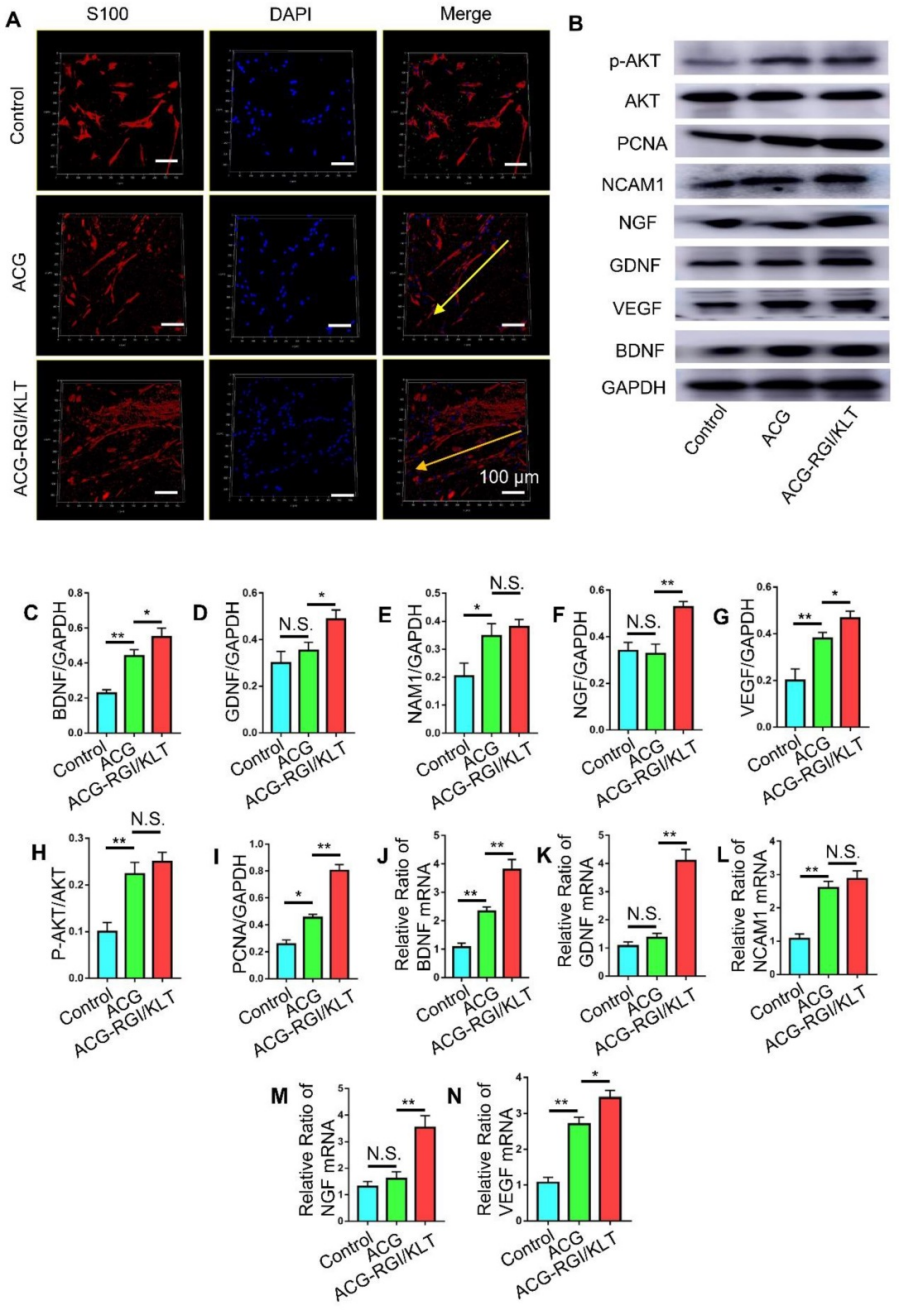
ACG-RGI/KLT regulated Schwann cells *in vitro*. (A) Images of cultured Schwann cells in the ACG, ACG-RGI/KLT, and control groups. Schwann cells were stained with anti-S100 (red) and DAPI (blue). (B) The expression levels of several markers reflecting the proliferation, adhesion, and secretory function of cells were examined by Western blotting (WB). (C-I) Statistical analysis of brain-derived neurotrophic factor (BNGF), nerve growth factor (NGF), glial cell-derived neurotrophic factor (GDNF), vascular endothelial growth factor (VEGF), neural cell adhesion molecule 1 (NCAM1), proliferating cell nuclear antigen (PCNA), and phosphorylated protein kinase B (p-AKT) protein levels, respectively. (K-N) qRT-PCR results indicated the relative expression levels of BNGF, NGF, GDNF, VEGF, and NCAM1. Data are presented as means ± standard error of the mean, *n* = 3 for each group. **p<0.01, * *p*<0.05.

Schwann cells play an important role in peripheral nerve regeneration, and guide the direction of regenerated axons. Schwann cells were inoculated onto the ACG, ACG-RGI/KLT, the control group (without any intervention). After 48 h, the Schwann cells in the ACG and ACG-RGI/KLT groups were oriented, while the cells in the control group were disordered. These observations indicated that ACG regulated the directional growth of Schwann cells (Figure 3A).

Next, we examined the effects of ACG-RGI/KLT on Schwann cells at the molecular level (Figure 3B). NGF, BGDN, GDNF, and VEGF are related to cell growth, while NCAM1 is associated with cell adhesion. The results of WB and qPCR analyses showed that NGF, BGDN, GDNF, VEGF, and NCAM1 expression levels were upregulated in Schwann cells on ACG-RGI/KLT (Figure 3C-G, J-N). ACG-RGI/KLT was also shown to increase the expression of PCNA (a cell proliferation marker) and p-AKT compared to the control group (Figure 3H-I). These observations indicated that ACG-RGI/KLT promoted the proliferation of Schwann cells by activating the PI3K/AKT signaling pathway, which is involved in the regulation of cell proliferation 48.

### ACG-RGI/KLT Promotes Sciatic nerve regeneration and remyelination *in vivo*

**Figure 4:**
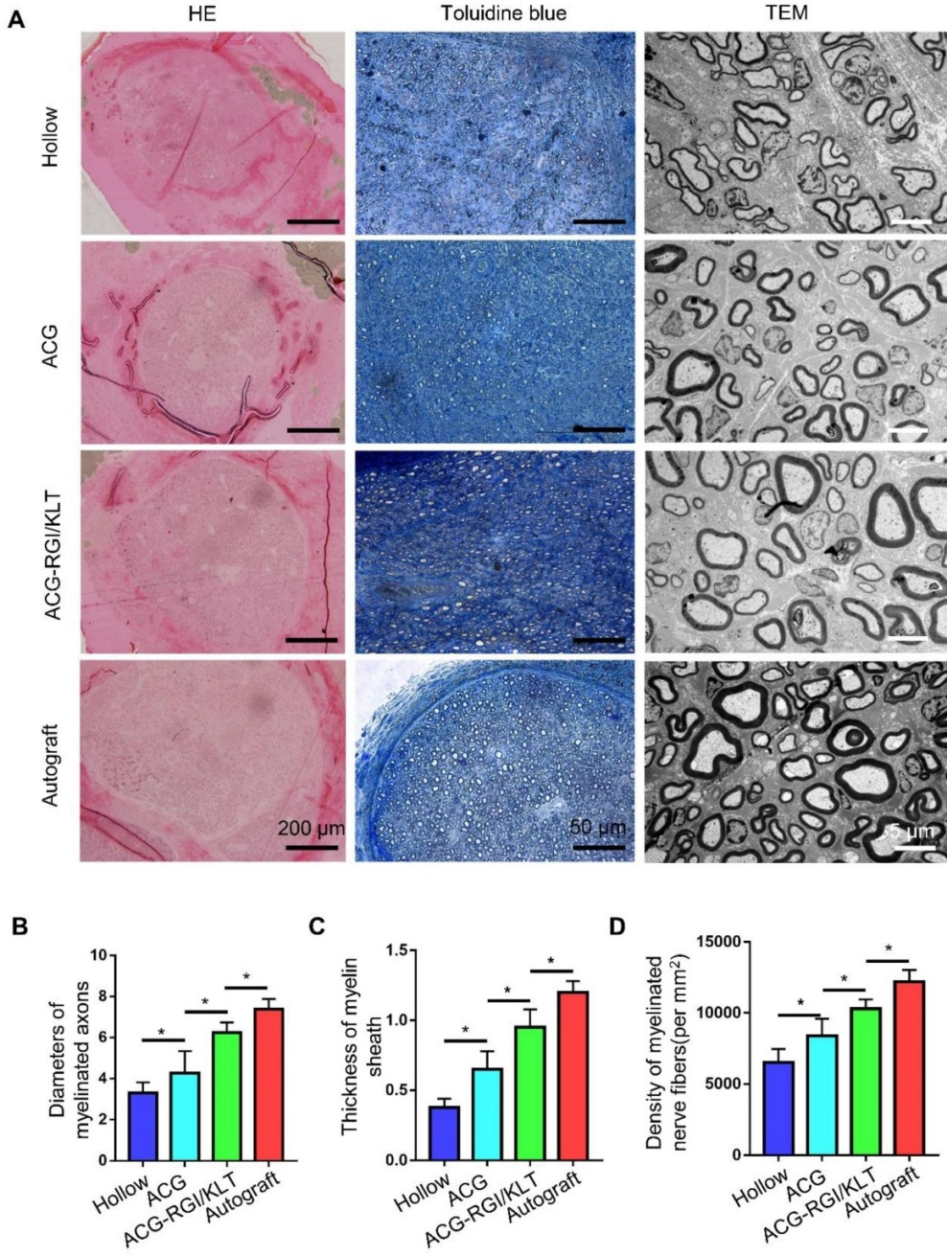
Evaluation of regenerated nerve fibers at 12 weeks after surgery. (A) Hematoxylin and eosin (HE) staining and toluidine blue staining in all groups. Transmission electron microscopy (TEM) images of the regenerated sciatic nerve. Statistical analysis of regenerated axons: calculation of the density of myelinated axons (B), diameters of myelinated axons (C), and thickness of the myelin sheath (D). All data are expressed as the mean ± standard error of the mean. *p<0.05.

At 12 weeks after the operation, the regenerated nerves in each group were removed after cardiac perfusion and subjected to osmium acid staining. The results of HE and toluidine blue staining showed that the number of regenerated nerve fibers in the ACG group was significantly higher than that in the hollow group (*p*<0.05), and the number of regenerated nerve fibers in the ACG-RGI/KLT group was higher than that in the ACG group (*p*<0.05). However, the highest number of regenerated nerve fibers was seen in the autologous nerve transplantation group (Figure 4A-B).

The diameter of regenerated myelinated nerve fibers and the myelin sheath thickness were evaluated by TEM (Figure 4A). The results showed that the regenerated myelinated nerve fibers in the hollow group were smallest in terms of diameter and thickness, due to the lack of support by scaffolds and growth factors (Figure 4C-D). The diameter and thickness of myelinated nerve fibers in the ACG group were greater than those in the hollow group (*p*<0.05). The diameter and thickness of regenerated myelinated nerve fibers in the ACG-RGI/KLT group were greater than those in the ACG group (*p*<0.05). However, there was still a difference between the ACG-RGI/KLT group and the autologous nerve transplantation group (p<0.05).

**Figure 5:**
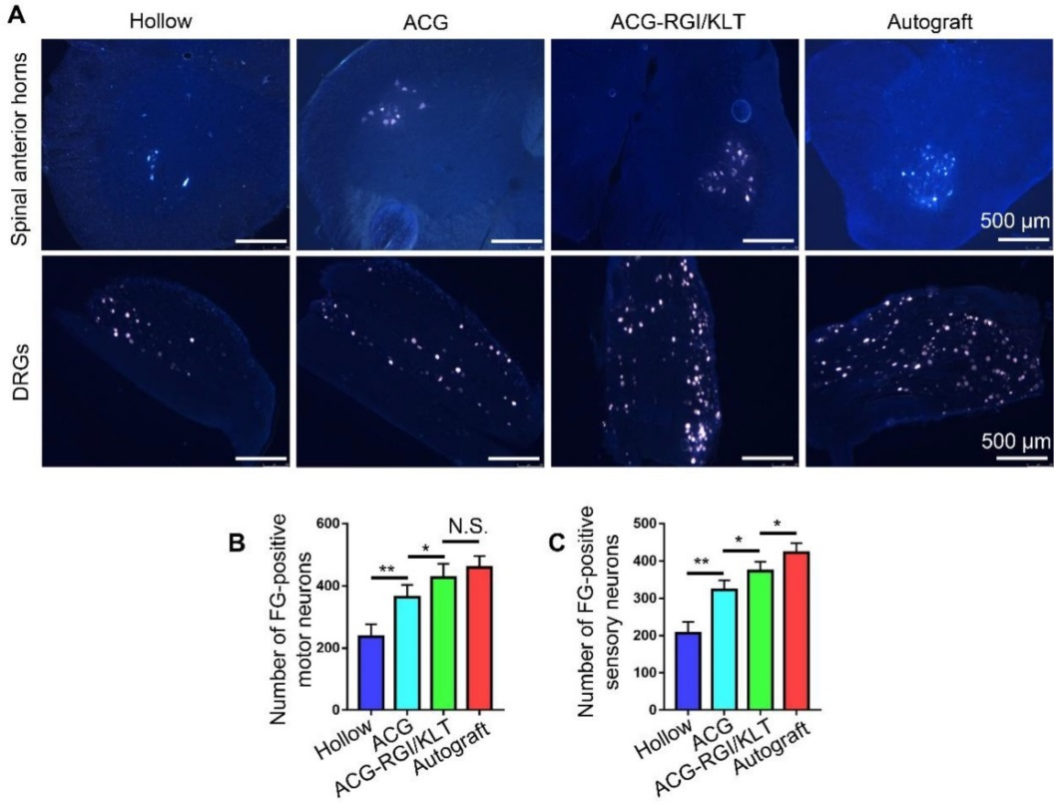
Fluoro-gold (FG) retrograde tracing in all groups. (A) Images of FG-labeled motor neurons in the spinal cord and sensory neurons in DRGs in all groups at 12 weeks after surgery. The average number of FG-positive motor neurons and sensory neurons in each group is shown in (B) and (C), respectively. All data are expressed as the mean ± standard error of the mean. * *p*<0.05, ** *p*<0.01.

The sciatic nerve is formed by the confluence of axons of motor neurons in the L4-6 anterior horn of the spinal cord and DRGs. Therefore, the number of superior neurons in the sciatic nerve is useful for evaluating regeneration of the sciatic nerve. The sciatic nerve was studied using the FG retrograde tracer at 12 weeks after the operation. After cardiac perfusion, the lumbar spinal cord was dissected out and cut into frozen sections for examination by fluorescence microscopy (Figure 5A). Statistical analysis showed that the number of motor neurons in the anterior horn of the spinal cord was higher in the ACG-RGI/KLT group than in the hollow and ACG groups (p<0.05), but was not significantly different from that in the autograft group (p>0.05) (Figure 5B). Similarly, the number of DRG neurons in the ACG-RGI/KLT group was higher than that in the ACG and hollow groups (p<0.05). However, the number of DRG neurons was highest in the autograft group (p<0.05) (Figure 5C).

### ACG-RGI/KLT Promotes motor function and electrical conduction recovery

**Figure 6:**
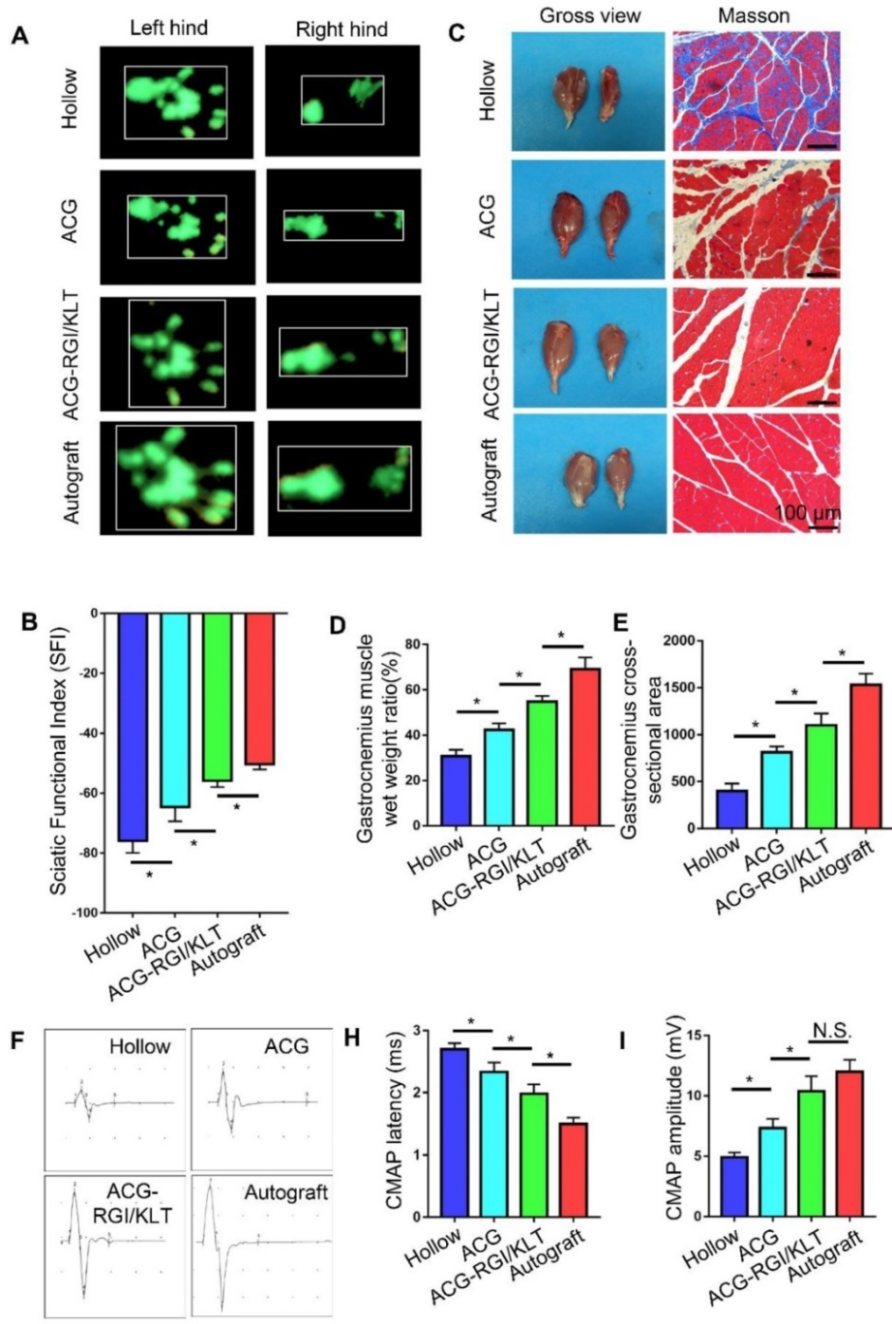
Recovery of motor function and electrical conduction in rats in all groups at 12 weeks after surgery. (A) Representative images of right hind paw (injured) and left hind paw (normal) footprints in all groups at12 weeks after the operation. (B) Statistical analysis of sciatic function index (SFI) values of all groups. All data are expressed as the mean ± standard error of the mean. *p<0.05, (*n* = 5). (C) Representative images of gastrocnemius muscles and Masson's trichrome staining images of transverse sections of gastrocnemius muscles. (D) Statistical analysis of the wet weight ratios of gastrocnemius muscles (injured leg vs. normal hind leg) (*n* = 5). (E) Statistical analysis of the mean cross-sectional area of gastrocnemius muscle fibers. All data are expressed as the mean ± standard error of the mean. * *p*<0.05. (F) Representative images of the complex muscle action potential (CMAP) of each group, as recorded by electrophysiological apparatus. Statistical analysis of CMAP amplitude (H) and latency (I) in each group. All data are expressed as the mean ± standard error of the mean. * *p*<0.05.

In rats, the denervated muscles begin to atrophy after sciatic nerve injury, which leads to limb paralysis. By recording rat footprints, the degree of reinnervation and functional recovery could be quantified. Twelve weeks after the operation, the gait of the rats in each group was examined by CatWalk XT 10.6 gait analysis (Figure 6A). The results showed that the SFI value of the ACG-RGI/KLT group was significantly lower than that of the hollow and ACG groups (*p*<0.05) (Figure 6B).

At 12 weeks after the operation, the nerve conduction function of all groups was tested. The amplitude and latency of gastrocnemius complex muscle action potential (CMAP) were measured electrophysiologically. The amplitude of CMAP is related to the number of muscle fibers in the neuromuscular junction, while the latency of CMAP is related to the thickness of the regenerated nerve myelin sheath. Therefore, recovery of neuromuscular function can be evaluated indirectly by measuring the amplitude and latency of CMAP. Figure 6F shows the CMAP of each group on electrophysiological examination. The CMAP latency in the ACG-RGI/KLT group was significantly lower than that in hollow and ACG groups (*p*<0.05), and greater than that in the autograft group (*p*<0.05) (Figure 6H). Similarly, the amplitude of CMAP in the ACG-RGI/KLT group was significantly greater than that in the hollow and ACG groups (*p*<0.05), but not significantly different from that in the autograft group (*p*>0.05) (Figure 6I). Taken together, these results indicated that ACG-RGI/KLT was effective for restoring nerve conduction function.

The neuromuscular system plays an important role in movement and, to a certain extent, the recovery of muscle function determines the recovery of motor function. At 12 weeks postoperatively, the gastrocnemius muscles of both hind limbs were cut in all groups. Atrophy of the gastrocnemius muscle was observed in all groups (Figure 6C). The wet weight ratio data also indirectly confirmed muscle atrophy. The wet weight ratio of the gastrocnemius muscle in the ACG-RGI/KLT group was significantly higher than that in the ACG and hollow groups (*p*<0.05) (Figure 6D), but still lower than that in the autograft group (*p*<0.05). The intensity of Masson's trichrome staining of gastrocnemius muscle cross-sections was significantly higher in the ACG-RGI/KLT group than in the hollow and ACG groups (*p*<0.05), and slightly lower than that in the autograft group (*p*<0.05) (Figure 6E).

### Mechanism by which ACG-RGI/KLT promotes nerve regeneration *in vivo*

**Figure 7:**
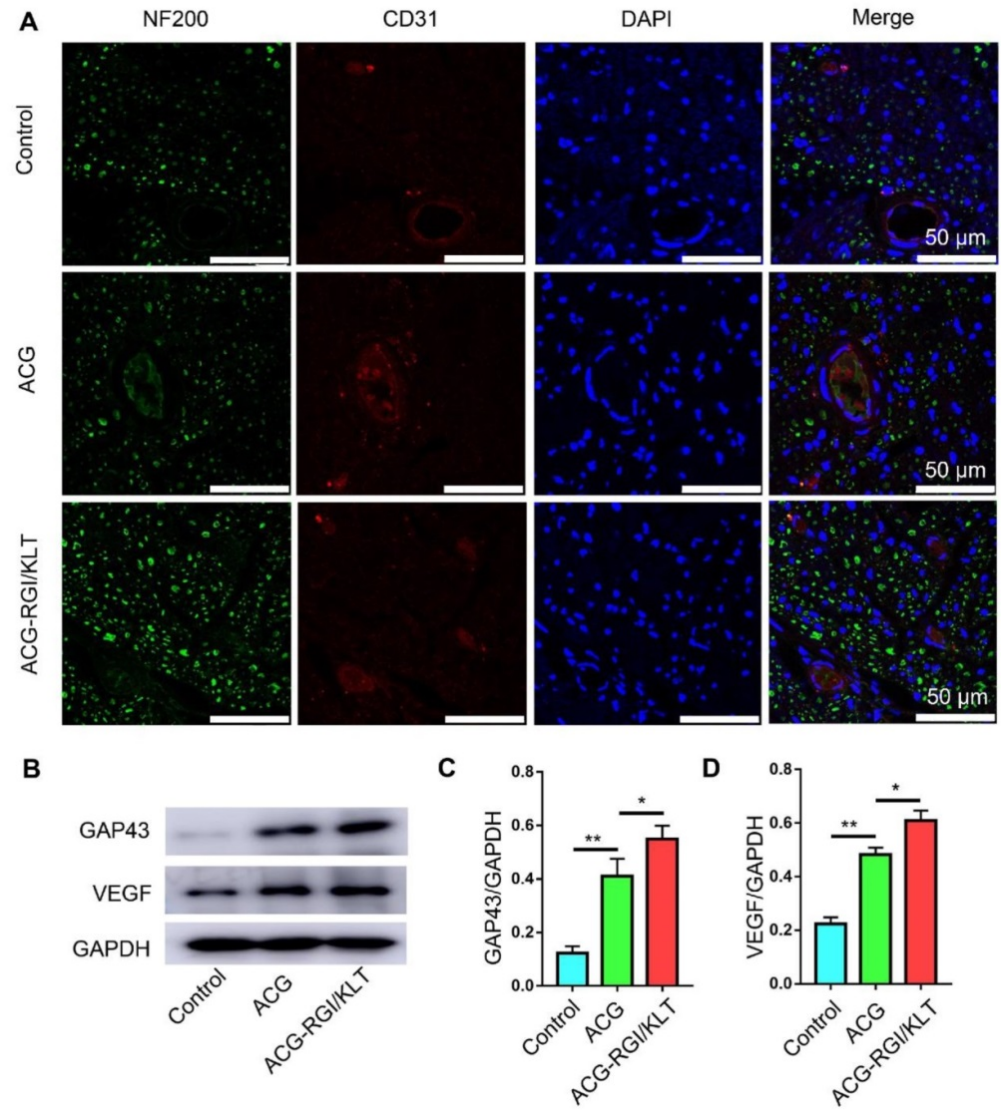
ACG-RGI/KLT promoted angiogenesis and regeneration of the sciatic nerve. (A) NF200 and CD31 immunofluorescence staining of regenerated nerves at 3 weeks after surgery. (B) WB of GAP43, CD31, and VEGF in regenerated nerves at 3 weeks after surgery. (C-E) Statistical analyses of GAP43, CD31, and VEGF, respectively. Data are presented as the mean ± standard error of the mean, *n* = 3 for each group. * *p*<0.05, ** *p*<0.01.

The results of CD31 and NF200 immunofluorescence analyses indicated significantly greater nerve fiber regeneration in the ACG-RGI/KLT group compared to the hollow and ACG groups at 4 weeks after the operation (Figure 7A). Similarly, the number of vessels in the ACG-RGI/KLT group was significantly greater than that in the ACG and hollow groups, indicating that ACG-RGI/KLT could promote angiogenesis. The results of WB also showed that ACG-RGI/KLT promoted VEGF expression compared to the hollow group and ACG group (*p*<0.05) (Figure 7B-C). The expression of GAP43, a protein highly expressed in the growth cones of regenerating neurons, was also examined in each group by WB and the results showed the same tendency as seen for VEGF (Figure 7B, D). Vascularization would thus promote the supply nutrients for nerve regeneration, and may have a synergistic effect with nerve regeneration.

## Discussion

Hierarchical alignment is a common phenomenon in nature and has specialized functions 49. The hierarchically aligned structure of nerve tissue is important for physiological and pathological repair. The peripheral nervous system can transmit nerve impulses quickly, in a manner dependent on the alignment of its anatomical structure 26, 42. Therefore, many nerve grafts are designed based on the aligned anatomical structure of peripheral nerve to repair peripheral nerve injury 6. In addition, the ECM provides a microenvironment suitable for nerve regeneration with a very low elastic modulus. Therefore, it is critical to develop novel nerve grafts with a hierarchically aligned structure, good biocompatibility and bioactivity, and mechanical properties compatible with the nerve ECM.

Hydrogels are widely used to repair various tissues because of their similar water content to human tissues. At present, porous hydrogels are widely used in bone and cartilage tissue repair because their three-dimensional network structure serves as a suitable microenvironment for cell adhesion, proliferation, and differentiation 50-56. However, hydrogels have an irregular and loose network structure, and are therefore not advantageous for peripheral nerve repair.

Electrospun nanofibers have great potential for application as regenerative medical scaffolds due to their structural similarity to the ECM. Electrospun nanofibers can guide cell morphology, migration, and differentiation. However, electrospinning usually produces planar membranes and it is difficult to form three-dimensional structures using this method. Therefore, we used liquid electrospinning and mechanical stretching methods to prepare irregular hydrogels as scaffolds with an aligned structure. These scaffolds can simulate the water content of nerve tissue and the anatomical structure of aligned peripheral nerves. Moreover, the elastic modulus of directional chitosan hydrogel is similar to that of nerve tissue. The liquid electrospinning technology used in this study is suitable for preparing aligned hydrogels from most macromolecules with fast cross-linking or self-assembly reactions, without the need to add toxic crosslinking agents during the preparation process. The whole reaction system can be controlled and the biological activity of the material can be fully realized.

However, controlling the alignment of hydrogel nanofibers remains a challenge. Kang et al. 57, 58 reported alginate hydrogel fibers based on microfluidic technology, in which alignment was achieved by extrusion through grooved microchannels. They reported that the growth of neurons was guided by the aligned fibers. However, the alignment was limited to the surface of the fibers. Zhang et al. 43 prepared long-distance polypeptide nanofiber hydrogels via thermally assisted self-assembly and mechanical shearing of amphiphilic polypeptide molecules. The aligned nanofibers effectively induced cell growth in a three-dimensional environment. However, this method is only suitable for certain peptide materials.

After peripheral nerve injury, Wallerian degeneration will occur at the distal end of the proximal stump. Schwann cells begin to proliferate and guide the regeneration of proximal axons. Fibrin cable formation occurring during the natural regenerative process occurring within a hollow NGC has been suggested to be critical for axon regeneration, by bridging the proximal and distal nerve stumps and guiding axonal outgrowth 59, 60. In the present study, aligned chitosan fibers were prepared as cables to guide neurite growth. In addition, neurotrophic factors, such as BDNF, CNTF, GDNF, NGF, NT3, and VEGF, are often used to regulate the local microenvironment for nerve regeneration 61, 62. However, the instability, short half-life, and high cost of neurotrophic factors limit their application. These problems can be avoided by using peptides that mimic the function of neurotrophic factors. In this study, two functional peptides, RGI and KLT, were used to mimic the functions of BDNF and VEGF, respectively. RGI and KLT, which are derived from functional fragments of BDNF and VEGF, respectively, grafted onto the surface of aligned chitosan fibers can provide a good microenvironment for nerve regeneration. BDNF can promote motor nerve regeneration to facilitate early recovery of motor function.

**Figure 8:**
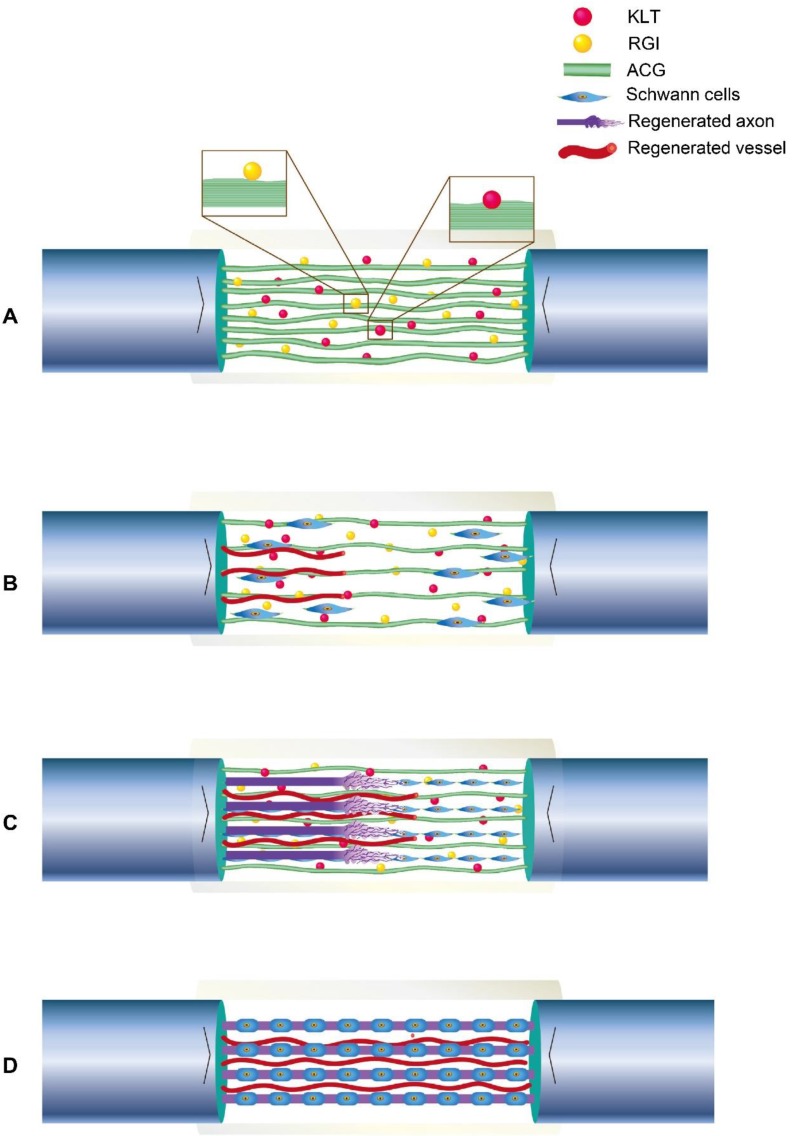
Illustration of the process of neural regeneration in the conduit. (A) ACG-RGI/KLT was used to bridge the nerve stumps. (B) Schwann cells migrated along the biological cables formed by ACG-RGI/KLT from the proximal and distal nerve stumps. (C) Newly regenerated axons sprouted along these cables. (D) Schwann cells wrapped around the axons to form myelin.

VEGF has been reported to promote tissue regeneration 63-65, and KLT can mimic the functional segment of VEGF. *In vitro*, we noted directional migration of Schwann cells in both the ACG-RGI/KLT and ACG groups. Moreover, Schwann cell proliferation was promoted by activation of the PI3K/AKT signaling pathway and, in turn, Schwann cells promoted the adhesion and secretion of a variety of neurotrophic factors, where such factors are essential for neurite growth. Parallel fibers are involved in the differentiation of stem cells and Schwann cells 42, 66, 67. For nerve defects > 10 mm, it is difficult for fibrin cables to support nerve regeneration 68. Therefore, in this study, the chitin conduit was filled with ACG to repair 15-mm sciatic nerve defects in rats. The process by which ACG-RGI/KLT likely promotes nerve regeneration is summarized in Fig. 8. The chitin conduit was shown to be an effective biodegradable material for peripheral nerve regeneration in previous studies 69. Chitin acts as the epineurium, providing mechanical support that can prevent stress due to locomotor activity in rats.

In addition, it was reported that chitooligosaccharides, which are degradation products of chitosan, induce macrophage infiltration and reconstruct the microenvironment at the injury site to promote nerve regeneration. Consistent with our *in vitro* results, the *in vivo* results also showed that nerve regeneration, myelination, conduction, target organ and motor function recovery were better in the ACG-RGI/KLT group versus the other groups, but autografting was still slightly superior.

## Conclusion

In this study, aligned chitosan combined with RGI/KLT was used to bridge nerve stumps, and rats lysosomes gradually degraded the chitosan fibers. The mechanism underlying the synergistic effects of RGI and KLT, and their optimal dosages, are still under investigation. The number of fibers added to the NGC lumen should be optimized because dense intraluminal matrices may impede the migration of regenerating axons and non-neuronal cells.

## Figures and Tables

**Table 1:** List of primer sequences used for qRT-PCR.

Primers	Forward	Reverse
GAPDH	ATGGTGAAGGTCGGTGTGAACG	TTACTCCTTGGAGGCCATGTAG
BDNF	AGTATTAGCGAGTGGGTC	GTTCCAGTGCCTTTTGTC
NGF	CAGCATGGTCGAGTTTTG	GATAGAAAGCTGCGTCCT
GDNF	CCGAAGATTATCCTGACC	CTCTCTCTTCGAGGAAGT
NCAM1	ATTGTCTGCTCCTCGGTC	GTTTGGGCTCAGTTTCTC
VEGF	AACTTCTACCCGTGCCTT	ACTTAGGTCAGCGTTTCC

## Abbreviations

ACG: Aligned chitosan fibers hydrogel
BDNF: Brain-derived neurotrophic factor
VEGF: vascular endothelial growth factor
NGCs: nerve guidance conduits
ECM: extracellular matrix
H&E: hematoxylin and eosin
PEO: poly (ethylene glycol)
STPP: sodium tripolyphosphate
SEM: scanning electron microscopy
TEM: transmission electron microscope
FG: Fluoro-gold
